# Erythropoietin Receptor Signaling Is Membrane Raft Dependent

**DOI:** 10.1371/journal.pone.0034477

**Published:** 2012-04-03

**Authors:** Kathy L. McGraw, Gwenny M. Fuhler, Joseph O. Johnson, Justine A. Clark, Gisela C. Caceres, Lubomir Sokol, Alan F. List

**Affiliations:** 1 Department of Malignant Hematology, H. Lee Moffitt Cancer Center, Tampa, Florida, United States of America; 2 Cancer Biology Ph.D. Program, University of South Florida, Tampa, Florida, United States of America; 3 Department of Gasteroenterology and Hepatology, Erasmus MC, University Medical Center Rotterdam, Rotterdam, The Netherlands; 4 Analytic Microscopy Core Facility, H. Lee Moffitt Cancer Center, Tampa, Florida, United States of America; 5 Department of Malignant Hematology, H. Lee Moffitt Cancer Center, Tampa, Florida, United States of America; Hungarian Academy of Sciences, Hungary

## Abstract

Upon erythropoietin (Epo) engagement, Epo-receptor (R) homodimerizes to activate JAK2 and Lyn, which phosphorylate STAT5. Although recent investigations have identified key negative regulators of Epo-R signaling, little is known about the role of membrane localization in controlling receptor signal fidelity. Here we show a critical role for membrane raft (MR) microdomains in creation of discrete signaling platforms essential for Epo-R signaling. Treatment of UT7 cells with Epo induced MR assembly and coalescence. Confocal microscopy showed that raft aggregates significantly increased after Epo stimulation (mean, 4.3±1.4(SE) vs. 25.6±3.2 aggregates/cell; p≤0.001), accompanied by a >3-fold increase in cluster size (p≤0.001). Raft fraction immunoblotting showed Epo-R translocation to MR after Epo stimulation and was confirmed by fluorescence microscopy in Epo stimulated UT7 cells and primary erythroid bursts. Receptor recruitment into MR was accompanied by incorporation of JAK2, Lyn, and STAT5 and their activated forms. Raft disruption by cholesterol depletion extinguished Epo induced Jak2, STAT5, Akt and MAPK phosphorylation in UT7 cells and erythroid progenitors. Furthermore, inhibition of the Rho GTPases Rac1 or RhoA blocked receptor recruitment into raft fractions, indicating a role for these GTPases in receptor trafficking. These data establish a critical role for MR in recruitment and assembly of Epo-R and signal intermediates into discrete membrane signaling units.

## Introduction

Erythropoietin (Epo) is the principal regulator of red blood cell production [1], [2]. Upon Epo binding to its cognate receptor (R), the Epo-R homodimerizes to initiate activation of the non-receptor tyrosine kinases JAK2 and Lyn, which in turn phosphorylate the receptor's cytoplasmic tail and the signal transducer and activator of transcription 5 (STAT5) [1], [2], [3]. Dimerization of phospho (P)-STAT5 enables its translocation to the nucleus and binding to target gene promoters, ultimately promoting the expansion, differentiation, and survival of red blood cell precursors [1], [2], [3]. The Epo signaling pathway is regulated by a balance of phosphatase and kinase activities [3]. Lyn kinase has been shown to enhance proliferation of erythroid progenitors by increasing colony forming capacity and promoting progenitor maturation [4], [5]. Loss of Lyn inhibits activation of STAT5 presumably through activation of negative regulatory phosphatases, such as Src homology domain-containing phosphatase-1 (SHP-1), SHP-2, and Src homology-2 domain-containing inositol 5-phosphatase 1 (SHIP-1) [6], [7]. Furthermore, association of Lyn with and phosphorylation of Epo-R and STAT5 promotes activation of downstream signaling [8]. Although the signaling cascade initiated by Epo and the balance of phosphatase and kinase activity has been well studied, the role of receptor localization in the plasma membrane and its effect on signal integrity has not been investigated.

The plasma membrane of hematopoietic cells contains sphingolipid and cholesterol enriched microdomains called lipid or membrane rafts [9], [10]. Lipid rafts represent hydrophobic, detergent-insoluble membrane fractions enriched in glycolipids and cholesterol. As a consequence, lipid rafts migrate to low density matrices upon gradient centrifugation allowing the isolation of raft membrane fractions and associated proteins [11], [12]. Lipid rafts are specialized membrane microdomains that cluster signaling intermediates to create focused signaling platforms that facilitate receptor-induced activation of signal transduction molecules. Rafts rapidly coalesce to form aggregates in response to cytokine stimulation or integrin engagement to optimize signal transduction [12], [13], [14], [15]. The clustering of rafts serves to expose proteins to a membrane environment enriched in components that amplify the signaling cascade, including kinases, scaffold and adaptor proteins, substrates as well as redistribution of regulatory phosphatases [12], [13], [14], [15]. Recent investigations have shown that raft microdomains have a critical role in T-cell receptor, c-kit and integrin signaling, protein trafficking, endocytosis, as well as many other diverse cellular functions [12], [16], [17], [18], [19], [20], [21]. In this study, we examined the role of lipid raft recruitment in Epo-R signaling, receptor interaction with signaling intermediates and Epo-R signal integrity.

## Results

### Epo induces raft formation and aggregation

**Figure 1:**
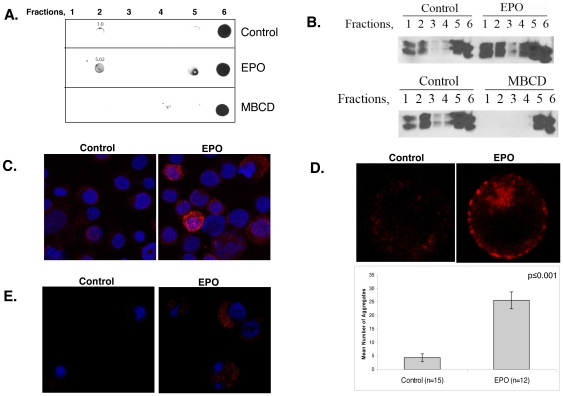
Epo stimulation induces raft formation and aggregation. (A) Dot blot detection of GM-1 in UT7 cell lysates in non-raft (fractions 5, 6) and raft fractions (fraction 2) with corresponding densitometry value in controls, and after Epo or MBCD treatment. Representative blot of at least three independent experiments. (B) Western immunoblot of Lyn in raft (R) (fractions 1–2) and non-raft (NR) fractions (fractions 4–6). Treatment with Epo increased Lyn kinase incorporation into raft fractions, whereas raft disruption by cholesterol depletion with MβCD precluded Lyn incorporation. Representative western of at least three independent experiments. (C) Immunofluorescence of UT7 cells showing an increase in raft (red) accumulation after Epo exposure. (D) Immunofluorescence of UT7 cells before and after Epo stimulation showing increased raft aggregates (red) in the plasma membrane and corresponding quantitation. (E) Immunofluorescence of primary erythroid bursts showing an increase in cellular membrane raft fluorescence intensity (red). Immunoflorescence experiments were repeated at least 3 times, representative micrographs displayed.

Lipid raft microdomains are characterized by their insoluble nature in non-ionic detergents as well as the presence of the constituent ganglioside GM-1 and double acylated proteins such as the Src-family kinase and Lyn kinase. We first investigated whether Epo affects membrane raft assembly or raft coalescence by assessing changes in membrane fraction distribution of GM-1 and Lyn kinase after Epo stimulation. Dot blot analysis of fractionated UT7 cell lysates revealed a greater than 5-fold increase of GM-1 in the detergent insoluble raft membrane fractions (fractions 1 and 2) after Epo exposure (Fig. 1A), accompanied by increased raft partitioning of Lyn kinase (Fig. 1B). To verify that the detergent insoluble fractions represented lipid rafts, we treated cells with a known membrane cholesterol chelating agent, methyl-β-cyclodextrin (MBCD), to disrupt raft integrity, and examined GM-1 and Lyn partitioning in membrane fractions. Treatment with MBCD abrogated partitioning of either GM-1 or Lyn into the detergent-insoluble membrane fractions, consistent with lipid raft distribution (Figs. 1A and B).

In T-lymphocytes, clustering of lipid rafts is an essential step in the formation of an immune synapse in response to antigen activation of the T-cell receptor [19]. To determine if Epo promotes raft coalescence, we quantitated changes in GM-1 labeled clusters after growth factor treatment. Raft accumulation in UT7 cells after Epo stimulation increased (Fig. 1C), accompanied by a significant increase in the mean number of raft aggregates (4.3±1.4 per cell in untreated controls compared to 25.6±3.2 aggregates per cell after Epo stimulation) (Fig. 1D; p≤0.001). The size of raft aggregates also increased after Epo treatment, with a 3.33±0.11 fold increase compared to unstimulated controls (p≤0.001). To verify that the observed changes in raft dynamics in UT7 cells extends to normal erythroid progenitors, we assessed raft assembly in bone marrow erythroid bursts derived from a normal donor. BFU-E were isolated by pipetting colonies grown in methylcellulose assays after 14 days incubation. Immunofluorescence staining for GM-1 (Fig. 1E) showed that mean raft fluorescence intensity in primary erythroid progenitors increased 58.4% from 72.79±14/cell in unstimulated cells to 115.27±14.22 after Epo treatment (p = 0.01).

### Epo-R co-localizes within lipid rafts

**Figure 2:**
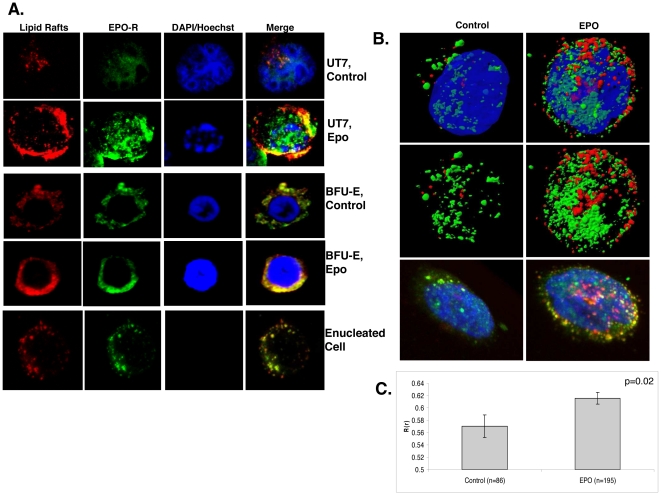
Epo-R co-localizes with lipid rafts. (A) Confocal immunofluorescence of cells untreated or after Epo stimulation, lipid rafts:red, Epo-R:green, DAPI/Hoechst:blue. Right panel is a merged image showing lipid raft and Epo-R co-localization (yellow). UT7 cells are shown in rows 1 and 2, while human primary burst forming units are shown in rows 3 and 4, followed by a maturing, enucleated erythroid precursor in row 5. (B) Three dimensional rendering of UT7 cells either untreated (left) or after Epo treatment (right). Top two rows display isosurfacing of the rafts (red), Epo-R (green), and nucleus (Dapi, blue). Dapi was removed from the middle row to further visualize association of the receptor with rafts in the second row of panels. The bottom row displays volume rendering of the same cells to illustrate membrane colocalization (yellow). (C) Quantitation of colocalization in human primary erythroid cells. Values represent mean ± SE. Immunofluorescence experiments were repeated at least 3 times, representative micrographs provided.

**Figure 3:**
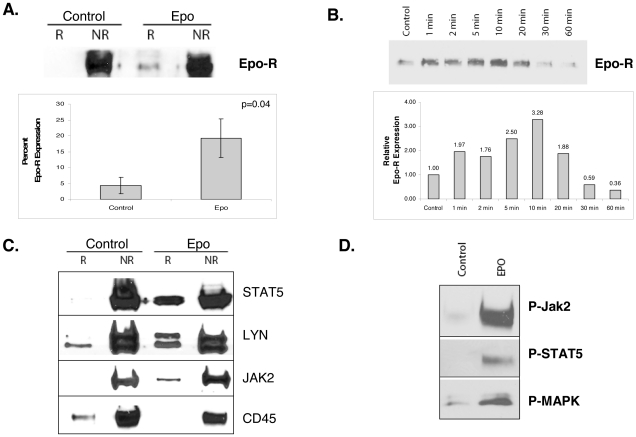
Epo stimulation recruits signal effectors into raft fractions. (A) Raft fractions (R) were separated from non-raft fractions (NR) and immunoblotted for Epo-R to investigate receptor translocation into rafts after Epo stimulation. Corresponding quantitation represents the mean ± SE of two independent experiments using four different Epo-R antibodies. (B) Raft fractions were isolated after stimulation with Epo at the indicated time points and immunoblotted for Epo-R. Results show that EpoR is recruited into rafts within 1 minute of Epo stimulation reaching maximum loading at 10 minutes, followed by gradual redistribution thereafter. Accompanying graphic quantitation of the representative experiment. (C) UT7 cells were starved overnight then treated with Epo for 10 min. After fractionation, the non-raft (NR) fractions and raft (R) fractions were pooled and immunoblotted for the indicated proteins. (D) Activated forms of Jak2, STAT5, and MAPK were also increased in the raft fractions after Epo stimulation. All westerns were repeated at least in duplicate.

Recruitment of the T-cell receptor into lipid rafts is a dynamic process, triggered by major histocompatability antigen engagement [12]. To determine if the Epo-R co-localizes within raft microdomains and is influenced by ligand engagement, we assessed Epo-R localization by confocal microscopy with and without Epo stimulation. Epo-R rapidly co-localized with GM-1 in UT7 cells after Epo stimulation (Fig. 2A, rows 1 and 2). Translocation of the Epo-R to membrane rafts after Epo treatment was also confirmed in primary bone marrow erythroid bursts (Fig. 2A, rows 3 and 4). In addition to immature erythroid progenitors such as burst forming units (BFU-E), colocalization of Epo-R in GM-1 raft clusters was also observed in more mature, enucleated erythroid cells after Epo stimulation (Fig. 2A, bottom row). To further illustrate the recruitment of receptor to the rafts, we utilized the power of 3D rendering. Figure 2B is a representative micrograph of an unstimulated (left) and stimulated (right) UT7 cell in which the number of rafts is increased (red) as well as the recruitment of the receptor (green) to these domains. The bottom row in Figure 2B utilizes volume rendering to emphasize the colocalization (yellow) of the rafts and receptor on the cell surface. We used the Pearson's coefficient to quantitate the percent of colocalization in primary BFU-E cells where there is a significant increase in colocalization after Epo stimulation (p = 0.02) (Fig. 2C). Epo-R membrane dynamics were further investigated by western blot analysis of membrane fractions from UT7 cell lysates isolated by gradient centrifugation. Raft (R) and non-raft (NR) fractions were pooled and separated by SDS-PAGE. Epo-R was not detected in lipid rafts from unstimulated cells, but was restricted to the membrane and cytosol fractions. After 10 min of Epo exposure, the receptor translocated into raft fractions (Fig. 3A), confirming that Epo-R ligand engagement triggers redistribution of the receptor to membrane raft microdomains. To confirm Epo-R specificity of antibody immuno-reactivity [22], [23], [24], receptor translocation was confirmed with several commercially available antibodies, including the Abcam mouse mAb (MM-0031-6G7) (Cambridge, MA), the Abcam goat polyclonal Epo-R antibody, and the monoclonal A82 Epo-R antibody generously provided by Amgen (Thousand Oaks, CA) [25], each of which confirmed our findings of ligand induced raft translocation. Densitometry analysis of 2 independent experiments using all 4 of the above mentioned antibodies is presented in Figure 3A. Based on recent investigations validating the specificity of the Santa Cruz Biotechnology (Santa Cruz, CA) Epo-R antibody (M-20), this antibody was used preferentially in subsequent experiments [26]. Furthermore, although Epo signaling is known to diminish after 10 minutes, we next investigated an extended range of intervals after Epo stimulation to discern the rapidity of receptor translocation into raft fractions (Fig. 3B). Epo-R was recruited into the raft fractions within 1 minute of growth factor exposure, reaching a peak at 10 minutes, followed by gradual redistribution that was completed by 60 minutes.

### Epo engagement initiates recruitment of signaling intermediates into lipid raft fractions

Because Epo-R was recruited into membrane rafts after growth factor stimulation, we investigated subcellular localization of corresponding signal effectors to determine if receptor translocation was coordinated with effector molecules to form discrete membrane platforms for receptor signaling. Immunostaining of membrane fractions for STAT5, JAK2, Lyn, and CD45 showed that Lyn and CD45 were constitutively localized in raft fractions in unstimulated cells, whereas JAK2 was absent with minimal detection of STAT5 (Fig. 3C). After Epo stimulation, both JAK2 and STAT5 (principal Epo signaling proteins) translocated into raft fractions accompanied by an increase in Lyn kinase. However, CD45, a receptor tyrosine phosphatase and key negative regulator of Epo-R signaling, was excluded from raft fractions and re-partitioned entirely into non-raft fractions (Fig. 3C). The differential localization of CD45 after Epo stimulation suggests that growth factor activation initiates a controlled process of raft assembly and aggregation favoring the recruitment of effector molecules supporting receptor signal transduction. Furthermore, we were able to show that the activated forms of both Jak2 and Stat5, as well as the alternative Epo signaling pathway, MAPK proteins, accompanied Epo-R in raft fractions after growth factor stimulation (Fig. 3D).

### Lipid rafts are required for Epo-R signaling

**Figure 4:**
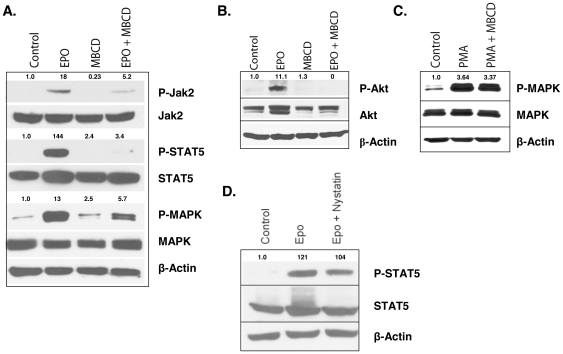
Raft integrity is necessary for Epo-induced signaling. (A) UT7 cells were starved for 2 h then pretreated with MBCD for 30 min and stimulated with 3 U/ml Epo for 10 min; lysates were immunoblotted with the indicated antibodies. (B) UT7/Epo cells were starved for 2 h then pretreated with MBCD for 30 min and stimulated with 3 U/ml Epo for 10 min. Lysates were immunoblotted with P-Akt. The findings show abrogation of Akt phosphorylation following MBCD pretreatment. (C) UT7 cells were pretreated with MBCD for 30 min, then stimulated with PMA for 30 min. (D) UT7 cells were starved for 2 h then pretreated with Nystatin for 30 min and stimulated with Epo for 10 min. Immunoblots for phospho-STAT5, STAT5, and β-actin antibodies with densitometry analysis. All westerns are representative of at least 2 independent experiments.

Given that Epo-R activation triggers formation of rafts enriched in signal effectors, we next investigated whether rafts are necessary for receptor signaling by way of raft microdomain disruption. Cholesterol depletion of UT7 cell membranes with methyl-β-cyclodextrin (MBCD) disrupted raft integrity and completely extinguished Epo induced phosphorylation of STAT5, the primary downstream transcription factor (Fig. 4A). To determine if secondary Epo signaling pathways were also affected by MBCD treatment, we probed UT7 cells for P-MAPK (mitogen-activated protein kinase). Indeed, pretreatment of cells with MBCD abrogated activation of MAPK with Epo stimulation. The PI3K/Akt pathway is not activated by Epo in UT7 cells, therefore, to investigate effects on this signaling pathway, we utilized the UT7/Epo cell line which displays Akt activation upon Epo stimulation (Fig. 4B). Pretreatment with MBCD completely extinguished activation of Akt by Epo, thereby confirming that all Epo signaling pathways are impaired by raft disruption. To verify that MBCD treatment only affected signaling pathways localized to lipid rafts, we treated UT7 cells with the cell permeable phorbol 12-myristate 13-acetate (PMA), which is not directly dependent on membrane receptor activation, and induces UT7 differentiation in part through the activation of MAPK. Pretreatment of UT7 cells with MBCD prior to PMA stimulation did not effect activation of MAPK as evidenced by enzyme phosphorylation (Fig. 4C). These data indicate that lipid raft integrity is essential for Epo-R signaling, whereas non-receptor or non-raft signaling pathways are preserved and independent of raft integrity. To confirm that abrogation of Epo-R/STAT5 signaling by MBCD is not specific to this compound, we repeated the above experiment using the cholesterol intercalating agent, nystatin, a less effective but alternative method to interfere with raft assembly and dynamics. Similar to our findings with MBCD, treatment with nystatin decreased STAT5 phosphorylation in response to Epo stimulation (Fig. 4D); providing further support for the importance of lipid rafts in Epo-R signal transduction.

### Raft disruption attenuates Epo-induced P-STAT5 induction in primary erythroid progenitors

**Figure 5:**
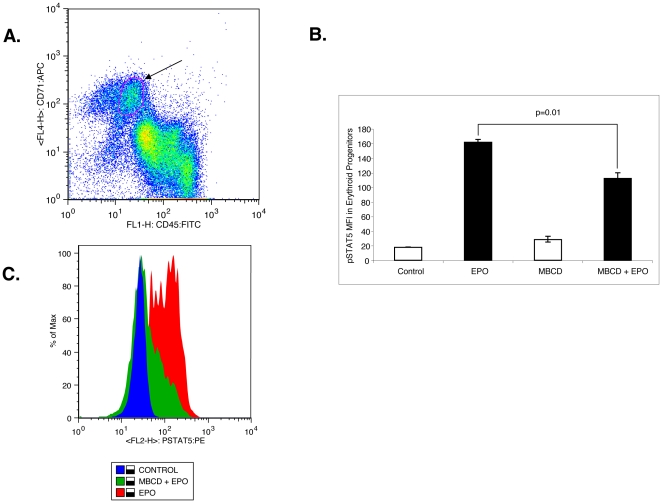
Cholesterol depletion attenuates Epo-induced STAT5 phosphorylation in primary erythroid progenitors. (A) Bone marrow mononuclear cells from a normal donor were isolated then stained with CD71:APC, CD45:FITC, and P-STAT5:PE. CD71^Hi^/CD45^dim^ cells representing erythroid progenitors were gated. (B) Graphic comparison of geometric mean florescence intensities, mean ± standard error from 3 independent experiments. (C) Representative flow histogram showing shift in phospho-STAT5 florescence intensity in primary erythroid progenitors treated with Epo with or without MβCD.

To confirm raft integrity is critical to Epo-R signaling in primary erythroid progenitors, we next assessed the effect of raft disruption by MBCD on Epo induced STAT5 phosphorylation by flow cytometry in bone marrow derived erythroid precursors from a normal donor. After a 2 h starvation, BM-MNCs were pretreated with MBCD either with or without Epo. Cells were permeabilized and stained with antibodies to CD71, CD45, and phospho-STAT5. Epo-responsive erythroid progenitors were identified by gating on the CD45 ^dim^ population of CD71+ cells (Fig. 5A), and phospho-STAT5 mean fluorescent intensity (MFI) was determined (Fig. 5B). Treatment with MBCD significantly decreased STAT5 phosphorylation in response to Epo stimulation (Fig. 5B; P = 0.01). Flow histograms show a marked shift consistent with a marked reduction in phospho-STAT5 MFI (Fig. 5C). These findings confirm that membrane raft integrity is critical to the fidelity of Epo-R signaling in primary erythroid precursors.

### Recruitment of Epo-R into lipid rafts is abrogated by Rac1 and RhoA inhibition

**Figure 6:**
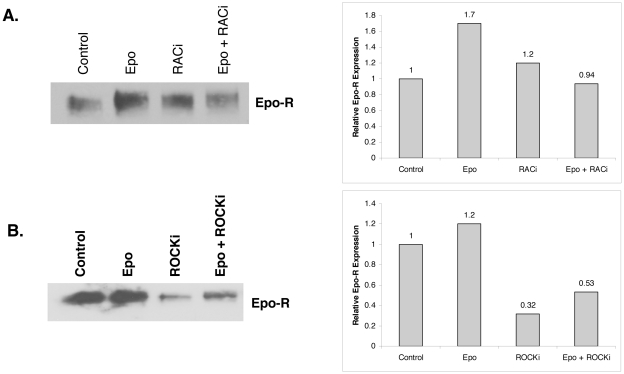
Recruitment of Epo-R into lipid rafts is dependent on Rac1 and RhoA GTPase activation. (A) Raft fractions were isolated from UT7 cells pretreated with 100 nM Rac1 inhibitor for 1 hr prior to Epo stimulation then immunoblotted for Epo-R with corresponding quantitation. (B) Raft fractions were isolated from UT7 cells pretreated with 100 uM ROCK inhibitor (Y-27632) for 1 h prior to Epo stimulation then immunoblotted for Epo-R with corresponding densitometry analysis. Westerns are representative of two independent experiments.

Rho GTPases are key regulators of intracellular actin dynamics, and are involved in T-cell receptor trafficking into lipid rafts upon receptor stimulation [19]. We therefore investigated whether GTPases were also involved in Epo-R recruitment into membrane rafts after Epo stimulation. UT7 cells were pretreated with a Rac1 inhibitor prior to Epo stimulation, demonstrating that inhibition of Rac1 suppressed recruitment of the receptor into raft fractions (Fig. 6A). We next investigated the effects of RhoA family GTPase inhibition by pretreating cells with the Rho-associated protein kinase, ROCK, inhibitor, Y-27632; again showing that Epo-R recruitment was blocked (Fig. 6B). These findings suggest that Rac1 and RhoA GTPase activation is critical in the redistribution of receptor into membrane fraction upon ligand binding.

## Discussion

To our knowledge, these are the first data to provide evidence that the Epo-R translocates into lipid raft microdomains of the plasma membrane upon ligand engagement (Fig. 2). Moreover, receptor recruitment into rafts appears necessary for Epo-R signal fidelity and consequent activation of STAT5. In unstimulated cells, the Epo-R resided largely in non-raft membrane fractions, which may serve to minimize the potential for ligand-independent interaction with signaling intermediates. Upon growth factor engagement, the receptor was recruited into lipid rafts accompanied by the incorporation of signaling effectors necessary to phosphorylate sites on the receptor tail and initiate signal transduction, including both the JAK2 and Lyn kinases, in addition to the principal downstream transcription factor, STAT5 (Fig. 3). Interestingly, CD45, a transmembrane protein tyrosine phosphatase that serves to extinguish receptor signaling by dephosphorylating JAK2 and the Epo-R, was constitutively localized within membrane rafts in unstimulated cells, whereas upon stimulation with Epo, re-partitioned exclusively into non-raft fractions. These dynamic changes in CD45 partitioning should serve to optimize receptor signaling upon ligand engagement, while restricting the potential for ligand-independent effector activation in the absence of the growth factor. Moreover, these ligand induced changes in the redistribution of the Epo-R and its effectors appear necessary for erythropoietin signal fidelity. Disruption of rafts by cholesterol depletion abrogated Epo-induced STAT5 phosphorylation in both UT7 cells and normal erythroid precursors (Figs. 4–5), whereas non-receptor initiated activation of MAPK by PMA remained intact. Intercalation of membrane cholesterol by nystatin treatment also attenuated Epo signaling, indicating that receptor integration into rafts is critical and perhaps obligatory for Epo-R signaling.

The subcellular mechanisms responsible for ligand induced changes in raft and receptor dynamics may involve G-protein controlled cytoskeletal changes. The dependence of Epo-R signaling on lipid raft recruitment and assembly is analogous to the changes observed in lymphocytes after ligation of the T-cell or B-cell receptors [19]. Within minutes of ligand engagement of the T-cell receptor, receptor subunits translocate into lipid rafts from their residence in non-raft membrane domains (Figure 3B). T-cell receptor re-distribution is controlled by G-protein coupled actin polymerization [19] involving activation of Rac GTPases, a hematopoietic specific member of the Rho superfamily that regulates the organization, dynamics and function of the actin cytoskeleton [27]. Conditional knock-out of *Rac2* was recently shown to block early stages of erythropoiesis in the bone marrow in murine models, suggesting that Rac2 may be a candidate molecular regulator of the observed Epo-induced changes in membrane dynamics [28], [29]. Our studies show that inhibition of either Rac1 or RhoA GTPases suppresses EpoR translocation into membrane raft domains. Defects in GTPase activation therefore could adversely affect receptor signaling in select pathologic conditions. In myelodysplastic syndromes, for example, Rac activation is impaired in neutrophils and CD34+ progenitors [30], accompanied by impaired lipid raft formation and a corresponding reduction in the generation of reactive oxygen species after fMLP stimulation in granulocyte-macrophage colony-stimulating factor primed neutrophils [31]. Abnormalities in raft assembly in erythroid progenitors might also underlie the previously described abnormalities in Epo-R signaling in MDS which warrants further investigation [32]. Overall, our findings indicate that ligand engagement of the Epo-R initiates dynamic changes in raft assembly and composition that bring the receptor and its effectors into spacial and temporal proximity within a discrete membrane compartment that facilitates activation of the signaling cascade. Development of strategies that enhance raft assembly and Epo-R incorporation may be an attractive strategy to improve erythropoiesis in hematologic disorders with impaired erythropoietic response.

## Materials and Methods

### Reagents and Antibodies

CD71:APC, P-STAT5(Y694):PE, and CD45:FITC conjugated antibodies used for flow cytometry and anti-CD45 used for western blotting were all purchased from BD Biosciences (San Jose, CA). STAT5, Lyn, Akt, P-Jak2, and Jak2 antibodies were purchased from Santa Cruz Biotechnology (Santa Cruz, CA). The principal Epo-R antibody used in this study was purchased from Santa Cruz Biotechnology (M-20). To confirm immuno-specificity of Epo-R localization (Fig. 3A) we also included Abcam mouse mAb (MM-0031-6G7), Abcam goat polyclonal, and Amgen (Thousand Oaks, CA) A82 Epo-R antibodies. ROCK inihibitor, Y-27632 dihydrochloride monohydrate, cholera toxin B (CTB) HRP conjugate, methyl-beta-cyclodextran, Nystatin, and PMA were purchased from Sigma-Aldrich (St. Louis, MO). P-MAPK, MAPK, and anti-P-STAT5(Y694) for westerns were purchased from Cell Signaling Technology (Danvers, MA). P-Akt, Alexa Fluor® 488 goat anti-rabbit IgG, and Vybrant® Lipid Raft Labeling Kit were ordered from Invitrogen (Carlsbad, CA). Recombinant human Epo (Epo) was purchased from Stemcell Technologies (Vancouver, BC, Canada). Rac1 Inhibitor was purchased from EMD Millipore (Billerica, MA).

### Cell Lines and Bone Marrow Cultures

The human leukemic cell line, UT7, was obtained from ATCC (Gaithersburg, MD). UT7 cells were maintained in α-MEM medium supplemented with 20% fetal bovine serum (FBS), 1% penicillin/streptomycin solution, and 5 ng/ml GM-CSF. UT7/Epo cells were maintained in IMDM medium supplemented with 10% FBS, 1% penicillin/streptomycin solution, and 1 U/mL Epo. After overnight starvation, cells were stimulated with Epo at a concentration of 3 U/mL. For Rac1 and ROCK inhibitor experiments, cells were pretreated for 1 h with 100 nM and 100 uM, respectively, before stimulation with Epo. Low-density mononuclear cell (MNC) fractions were isolated from heparinized bone marrow aspirates from healthy volunteers purchased from Lonza Walkersville Inc. (Walkersville, MD) using standard density gradient centrifugation over Ficoll-Paque Plus (GE Healthcare, Little Chalfont, United Kingdom), followed by washing and resuspension in Iscove's Modified Dulbecco Medium (IMDM) supplemented with 10% fetal bovine serum, penicillin, and streptomycin.

Erythroid progenitors at the burst-forming unit–erythroid (BFU-E) stage of differentiation were grown in cytokine-defined IMDM, similar to previous studies [33]. Briefly, 2×10^5^ MNC per mL were plated in Complete Methocult® medium (StemCell Technologies, Vancouver, BC, Canada) supplemented with 10% FBS and 3 U/mL erythropoietin. Plates were incubated at 37°C in a 5% CO_2_ air mixture in a humidified incubator for 14 days. BFU-E were identified using an inverted microscope, aspirated by pipette, washed twice in PBS then resuspended in IMDM for immunofluorescence studies.

### Immunoblotting

Cells were starved in 0.5% FBS containing medium for 2 h prior to 30 min pre-incubation with 10 mM MBCD or 50 µg/ml nystatin, or stimulation with 3 U/ml Epo (10 min) or 100 ng/ml PMA (30 min). For RAC and ROCK inhibitor experiments, cells were pretreated for 1 h prior to Epo stimulation. Cells were washed 3× in cold PBS and lysed in 1× RIPA buffer containing 250 µM NaVO_4_, 2 µg/ml aprotinin, 2 µg/ml leupeptin, 0.2 µg/ml pepstatin A, and 500 µM PMSF. Sample buffer was added to cell lysates and 100 µg of protein was separated using SDS-PAGE. Proteins were transferred to PVDF membranes and immunoblotted with the indicated antibodies. Membranes were developed using ECL or ECL Plus according to manufacturer's protocols (GE Healthcare, Piscataway, NJ).

### Flow Cytometry

Bone marrow from a normal donor was purchased from Lonza (Walkersville, MD). BM-MNCs were isolated using Ficoll-Paque PLUS (GE Healthcare, Piscataway, NJ) and starved for 2 h in 0.5% FBS containing IMDM medium. The cells were then pretreated with 10 mM MBCD for 30 min and stimulated with 3 U/ml Epo for 10 min. They were immediately washed 3× in cold Staining Buffer (BD Biosciences San Jose, CA), fixed for 10 min at 37°C in Cytofix (BD), then permeabilized for 30 min on ice with Perm Buffer III (BD). Cells were stained with CD71:APC, CD45:FITC, and P-STAT5:PE conjugated antibodies. Cells were washed with Staining Buffer and analyzed on a FACScalibur flow cytometer. Primitive erythroid cells were captured in CD71^Hi^ and CD45^Dim^ gated population.

### Lipid Raft Isolation

Lipid Rafts were isolated as previously described [11], [31]. Briefly, UT7 cells were washed 2× with cold PBS then lysed in 0.75% Triton X-100 in TNE Buffer [TNE buffer composed of 25 mM Tris pH7, 150 mM EDTA, 1 mM DTT, 150 mM NaCl, and 1 Complete EDTA-free protease inhibitor tablet from Roche (Indianapolis, IN) per 20 ml buffer]. Cells were passed through a 27G needle several times and incubated on ice for 5 min. Two hundred microliters of lysate were mixed with 400 µL of 60% Optiprep (Sigma-Aldrich, St. Louis, MO) and pipetted into an ultracentrifuge tube. Decreasing percentages of Optiprep (35%, 30%, 25%, 20%, and 0%) were loaded on top of each other and the tubes were spun at 20000 rpm for 20 h in a Beckman Coulter (Fullerton, CA) Optima L-90K ultracentrifuge. Fractions were pipetted off one by one and used for dot and western blotting.

### Dot Blots

Five or ten microliters of fractionated cell lysates were pipetted directly onto nitrocellulose membrane. The membranes were allowed to dry then washed briefly in PBS. They were then blocked in 0.3% Tween20 PBS for 30 min and incubated in cholera toxin B:HRP conjugated antibody overnight. The blots were washed 3× in 0.3% Tween20 PBS and developed with ECL.

### Immunofluorescence

Starved UT7 cells (0.5% FBS supplemented α-MEM medium) were stained with Vybrant® Lipid Raft Labeling Kit according to manufacturer's protocol, treated with 3 U/ml EPO for 10 min at 37°C and fixed with Cytofix (BD Biosciences San Jose, CA) for 10 m at 37°C. Cells were then cytospun and stained with Epo-R antibody at a 1∶50 dilution for 1 hr at 37°C, washed in PBS and stained 1∶500 with Alexa Fluor® 488 goat anti-rabbit IgG for 1 hr at 37°C. Cells were then mounted using ProLong® AntiFade reagent with DAPI (Invitrogen, Carlsbad, CA) and cover slip placed on top. Micrographs were taken using a Leica TCS SP5 AOBS Laser Scanning Confocal microscope (Leica Microsystems, Germany). BFU-E colonies isolated from progenitor cultures from a normal donor were washed 2× then starved in 0.5% FBS supplemented IMDM medium for 2 h. They were then stained with Epo-R and Alexa Fluor® 488 goat anti-rabbit IgG as above. The cells were then washed and stained with Vybrant® Lipid Raft Labeling Kit according to manufacturer's protocol. Cells were resuspended in 1 ml medium and stained with 1 µg/ml Hoechst stain (Invitrogen, Carlsbad, CA). Micrographs of the untreated cells were taken by confocal microscopy then 3 U/ml of Epo was added to the plate and micrographs from stimulated cells were taken 5–20 min after Epo treatment.

### Immunofluorescence Image Analysis

Photomicrographs were obtained using a Leica TCS SP5 AOBS laser scanning confocal microscope at zoom through a 20×/0.5NA or 63×/1.40NA Plan Apochromat oil immersion objective lens (Leica Microsystems, Germany). 405 Diode, Argon 488, and HeNe 543 or 594 laser lines were applied to excite the flurophores and tunable emissions were used to minimize crosstalk between fluorochromes. Gain, offset, and pinhole settings were identical for all samples within the treatment group. Image sections were collected at either 0.2 µm (for 3D reconstructions) or at 0.5 µm were captured with photomultiplier detectors and maximum projections were prepared with the LAS AF software version 2.1.0 (Leica Microsystems, Germany). In some cases, 4× zoom was applied when acquiring images. Intensity and aggregate analysis were performed using Image Pro Plus version 6.2 (Media Cybernetics, Inc., Silver Springs, Maryland). Identical threshold settings and measurement parameters were used to generate the mean intensity and area data. Aggregates were defined as an object within the cell that has an intensity value of at least 20 and an area between 3 and 600 pixels. Three dimensional isosurface renderings were prepared with Imaris software version 5.5.3 (Bitplane Inc., Zurich, Switzerland).

### Statistical analysis

Numerical data are expressed as mean ± standard error of the mean. Statistical analyses were performed using the Student t test (2-tailed for equal variances). P values<0.05 were considered significant. Pearson's correlation analysis for colocalization was performed using Definiens Developer version 1.5 (Definiens AG, Munich, Germany).
